# Different modes of synaptic and extrasynaptic NMDA receptor alteration in the hippocampus of P301S tau transgenic mice

**DOI:** 10.1111/bpa.13115

**Published:** 2022-09-04

**Authors:** Rocío Alfaro‐Ruiz, Carolina Aguado, Alejandro Martín‐Belmonte, Ana Esther Moreno‐Martínez, Jesús Merchán‐Rubira, Félix Hernández, Jesús Ávila, Yugo Fukazawa, Rafael Luján

**Affiliations:** ^1^ Synaptic Structure Laboratory, Instituto de Investigación en Discapacidades Neurológicas (IDINE), Departamento de Ciencias Médicas, Facultad de Medicina Universidad Castilla‐La Mancha, Campus Biosanitario Albacete Spain; ^2^ Centro de Biología Molecular Severo Ochoa CSIC‐UAM Madrid Spain; ^3^ Centro de Investigación Biomédica en Red sobre Enfermedades Neurodegenerativas ISCIII Madrid Spain; ^4^ Division of Brain Structure and Function, Faculty of Medical Science University of Fukui Fukui Japan; ^5^ Life Science Innovation Center University of Fukui Fukui Japan; ^6^ Present address: Pharmacology Unit, Department of Pathology and Experimental Therapeutics Faculty of Medicine and Health Sciences, Institute of Neurosciences, University of Barcelona 08907 L'Hospitalet de Llobregat Spain

**Keywords:** AD mouse model, Alzheimer disease, electron microscopy, freeze‐fracture, hippocampus, immunohistochemistry, NMDA receptors

## Abstract

*N*‐methyl‐d‐aspartate receptors (NMDARs) are pivotal players in the synaptic transmission and synaptic plasticity underlying learning and memory. Accordingly, dysfunction of NMDARs has been implicated in the pathophysiology of Alzheimer disease (AD). Here, we used histoblot and sodium dodecylsulphate‐digested freeze‐fracture replica labelling (SDS‐FRL) techniques to investigate the expression and subcellular localisation of GluN1, the obligatory subunit of NMDARs, in the hippocampus of P301S mice. Histoblots showed that GluN1 expression was significantly reduced in the hippocampus of P301S mice in a laminar‐specific manner at 10 months of age but was unaltered at 3 months. Using the SDS‐FRL technique, excitatory synapses and extrasynaptic sites on spines of pyramidal cells and interneuron dendrites were analysed throughout all dendritic layers in the CA1 field. Our ultrastructural approach revealed a high density of GluN1 in synaptic sites and a substantially lower density at extrasynaptic sites. Labelling density for GluN1 in excitatory synapses established on spines was significantly reduced in P301S mice, compared with age‐matched wild‐type mice, in the stratum oriens (so), stratum radiatum (sr) and stratum lacunosum‐moleculare (slm). Density for synaptic GluN1 on interneuron dendrites was significantly reduced in P301S mice in the so and sr but unaltered in the slm. Labelling density for GluN1 at extrasynaptic sites showed no significant differences in pyramidal cells, and only increased density in the interneuron dendrites of the sr. This differential alteration of synaptic versus extrasynaptic NMDARs supports the notion that the progressive accumulation of phospho‐tau is associated with changes in NMDARs, in the absence of amyloid‐β pathology, and may be involved in the mechanisms causing abnormal network activity of the hippocampal circuit.

## Graphical abstract

*Differential alteration of synaptic versus extrasynaptic NMDARs in P301S mice*. Reduced density of synaptic NMDARs in excitatory synapses in the hippocampus of P301S mice at 10 months. NMDARs at excitatory synapses established on pyramidal cell spines and interneurons are significantly reduced in the CA1 region of the hippocampus compared to age‐matched wild type controls. However, extrasynaptic NMDARs were unaltered, thus producing an imbalance between synaptic and extrasynaptic NMDARs that may contribute to the pathological alterations of P301S mice.

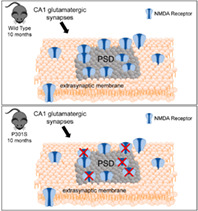

## 1 INTRODUCTION

Glutamate is the main excitatory neurotransmitter in the brain acting on both ionotropic and metabotropic receptors [ 1 ]. The *N*‐methyl‐d‐aspartate (NMDA)‐type glutamate receptors comprise a class of ionotropic glutamate receptors that play a central role in synaptic plasticity, circuit development, memory formation and neuronal survival [ 1 ]. However, overactivation of *N*‐methyl‐d‐aspartate receptors (NMDARs) causes excitotoxicity to promote neuronal death that takes place in the pathophysiology of neurodegenerative disorders, including Alzheimer disease (AD) [ 2 ]. The impairments in memory and cognition characteristic of AD, can be correlated to neuropathological features that include the formation of senile plaque of amyloid‐β (Aβ) and neurofibrillary tangles (NFT) of phospho‐tau, and synapse loss in the hippocampus [ 3 ]. However, decreased synaptic density is the strongest anatomical correlate of the degree of clinical impairment [ 4 ].

Molecular cloning revealed seven different NMDAR subunits: the GluN1 subunit, four GluN2 subunits (GluN2A, GluN2B, GluN2C and GluN2D) and two GluN3 subunits (GluN3A and GluN3B) [ 5 ]. Functional NMDARs are heterotetramers composed by two GluN1 obligatory subunits and two regulatory subunits: GluN2 (A–D) or GluN3 (A–B) [ 6, 7 ]. The GluN1 subunit exists in several alternatively spliced forms [ 1 ] and is expressed ubiquitously throughout the brain, whereas GluN2 subunits show both regional and developmental variations in animals, with GluN2A and GluN2B being the major regulatory subunits in the hippocampus [ 8, 9 ]. These two subunits confer different pharmacological and biophysical properties to functional NMDARs and are believed to play a determining role in synaptic plasticity [ 10 ].

The abundance and molecular composition of NMDARs by specific neuron types is central to our understanding of synaptic transmission and synaptic plasticity. NMDARs present in excitatory synapses established on dendritic spines of pyramidal cells are responsible for the entry of Ca^2+^ and are required for the induction of long‐term changes in synaptic efficacy [ 11 ]. In interneurons, NMDARs play a role in long‐term changes in synaptic efficacy and participate in the synaptic activation of interneurons [ 12 ]. Consequently, any alteration in the number and density of NMDARs could contribute to the synaptic and memory deficits that are associated with AD. The application of Aβ decreases cell surface expression of NMDARs and spine density through a pathway that requires NMDARs [ 13, 14 ]. However, the molecular mechanisms of tau‐mediated neurotoxicity are unclear. In AD, tau becomes hyperphosphorylated and mislocalises to pre‐ and post‐synaptic compartments of neurons, thus contributing to synaptic dysfunction and affects NMDAR trafficking and organisation [ 15, 16 ]. The way the molecular organisation of synapses is affected by tau mislocalisation in AD is unknown.

Immunoelectron microscopic approaches confirmed the presence of NMDARs in excitatory synapses on dendritic spines and interneuron dendrites [ 17, 18, 19 ], and at extrasynaptic compartments albeit at a lower density [ 20 ]. The distribution of synaptic versus extrasynaptic NMDARs has emerged as an important parameter contributing to neuronal dysfunction in neurodegenerative diseases including AD [ 21 ]. The possible alteration in the NMDAR content of different excitatory synapses and extrasynaptic compartments in tau models of AD has not been explored. Therefore, in the present study, we used P301S mice, a commonly used tauopathy model with several AD‐relevant features [ 22 ], to investigate whether hyperphosphorylated tau is associated with changes in the expression and subcellular localisation of the obligatory NMDAR subunit, GluN1, in the hippocampus. Here, we show convincing evidence for a reduction in synaptic NMDARs, but not extrasynaptic NMDARs, in pyramidal cells and interneurons of the hippocampal Cornu Ammonis (CA)1 field in P301S mice.

## 2 MATERIALS AND METHODS

### 2.1 Animals

We used *female* transgenic mice P301S for the human Tau gene and wild‐type (WT) control littermates. The P301S mouse model, obtained from Jackson Laboratory (B6;C3‐Tg(Prnp‐MAPT*P301S)PS19Vle/J), carries a mutant (P301S) human MAPT gene encoding T34‐tau isoform (1N4R) driven by the mouse prion‐protein promoter (Prnp) on a B6C3H/F1 genetic background. For analysis, we selected animals of 3 and 10 months of age. P301S mice of 3 months old were characterised by initial signs of the pathology in the hippocampus. For instance, hippocampal synapse loss and impaired synaptic function were detected at this age before fibrillary tau tangles emerged, but brain atrophy, neuronal death in the hippocampus or microglial activation were not detected [ 22 ]. P301S mice of 10 months old are characterised by widespread NFT accumulation in the hippocampus, impaired memory, spatial learning and long term potentiation (LTP), impaired synaptic function, prominent neuronal death, microglial activation and around 30% volume reduction in the hippocampus [ 22 ]. A total of 6 female mice aged 3 months (*n* = 3 for WT, *n* = 3 for P301S used for histoblotting) and 12 mice aged 10 months (*n* = 3 for WT, *n* = 3 for P301S used for histoblotting; and *n* = 3 for WT, *n* = 3 for P301S used for immunoelectron microscopy) were analysed. All mice were housed at the ‘Centro de Biología Molecular Severo Ochoa’ animal facility. Mice were housed four per cage with food and water available ad libitum and maintained in a temperature‐controlled environment on a 12/12 h light–dark cycle with light onset at 07:00 h. Animal housing and maintenance protocols followed the guidelines of Council of Europe Convention ETS123, recently revised as indicated in Directive 86/609/EEC. Animal experiments were performed under protocols (P15/P16/P18/P22) approved by the Institutional Animal Care and Utilization Committee (Comité de Ética de Experimentación Animal del CBM, CEEA‐CBM, Madrid, Spain).

For histoblotting, the animals were deeply anaesthetised by intraperitoneal injection of ketamine/xylazine 1:1 (0.1 ml/kg b.w.), the brains were quickly frozen in liquid nitrogen and stored at −80°C. For the sodium dodecylsulphate‐digested freeze‐fracture replica labelling (SDS‐FRL) technique, animals were anaesthetise with sodium pentobarbital (50 mg/kg, i.p.) and perfused transcardially with 25 mM phosphate‐buffered saline (PBS) for 1 min, followed by perfusion with 2% (*w*/*v*) paraformaldehyde in 0.1 M phosphate buffer (PB) for 12 min. After perfusion, brains were removed from the skull and the hippocampi were dissected and cut into coronal slices (130 μm) using a Microslicer (Dosaka, Kyoto, Japan) in 0.1 M PB.

### 2.2 Antibodies and chemicals

For histoblot and SDS‐FRL, we used a mouse monoclonal antibody against the GluN1 subunit of NMDA receptor (MAB363; Millipore Bioscience Research Reagents). This antibody was directed against a fusion protein corresponding to amino acid residues 660–811, representing the extracellular loop between transmembrane regions III and IV of the GluN1 subunit [ 23 ]. The characteristics and specificity of GluN1 were characterised previously in the literature [ 23 ].

The secondary antibodies used were as follows: goat anti‐mouse IgG‐horseradish peroxidase (1:2000; Santa Cruz Biotechnology, Santa Cruz, CA), alkaline phosphatase (AP)‐goat anti‐mouse IgG (H + L), anti‐mouse IgG conjugated to 10 nm gold particles (1:100; British BioCell International, Cardiff, UK).

### 2.3 Histoblotting

The regional distribution of GluN1 was analysed in mouse brains, using the histoblot technique [ 24 ]. Briefly, horizontal cryostat sections (10 μm) from mouse brain (coordinates: from interaural: 6.16 mm/bregma: −3.84 mm to interaural: 4.36 mm/bregma: −5.64 mm) were overlapped with nitrocellulose membranes moistened with 48 mM Tris‐base, 39 mM glycine, 2% (*w*/*v*) sodium dodecyl sulphate (SDS) and 20% (*v*/*v*) methanol for 15 min at room temperature (~20°C). After blocking in 5% (*w*/*v*) non‐fat dry milk in PBS for 1 h, nitrocellulose membranes were treated with DNase I (5 U/ml), washed and incubated in 2% (*w*/*v*) SDS and 100 mM β‐mercaptoethanol in 100 mM Tris–HCl (pH 7.0) for 60 min at 45°C to remove adhering tissue residues. After extensive washing, the blots were reacted with affinity purified anti‐GluN1 antibodies (0.5 mg/ml) in blocking solution overnight at 4°C. The bound primary antibodies were detected with AP‐conjugated anti‐mouse IgG secondary antibodies [ 24 ]. A series of primary and secondary antibody dilutions and incubation times were used to optimise the experimental conditions for the linear sensitivity range of the AP reactions. To compare the expression levels of NMDARs between the two genotypes (WT and P301S) and ages (3 and 10 months), all nitrocellulose membranes were processed in parallel, and the same incubation time for each reagent was used for the antibody. Digital images were acquired by scanning the nitrocellulose membranes using a desktop scanner (HP Scanjet 8300). Image analysis and processing were performed using the Adobe Photoshop software (Adobe Systems, San Jose, CA, USA) as described previously in the literature [ 25 ].

### 2.4 SDS‐digested freeze‐fracture replica labelling

Immunohistochemical reactions at the electron microscopic level were carried out using the SDS‐FRL method as described earlier in the literature [ 26 ]. Briefly, we trimmed hippocampal slices containing the CA1 field and immersed them in graded glycerol of 10%–30% (*v*/*v*) in 0.1 M PB at 4°C overnight. Slices were frozen using a high‐pressure freezing machine (HPM010, BAL‐TEC, Balzers, Liechtenstein). Slices were then fractured into two parts at −120°C and replicated by carbon deposition (5 nm thick), platinum (60° unidirectional from horizontal level, 2 nm), and carbon (15 nm) in a freeze‐fracture replica machine (BAF060; BAL‐TEC, Balzers, Liechtenstein). Replicas were transferred to 2.5% (*w*/*v*) SDS and 20% (*w*/*v*) sucrose in 15 mM Tris buffer (pH 8.3) for 18 h at 80°C with shaking to dissolve tissue debris. The replicas were washed three times in 50 mM Tris‐buffered saline (TBS, pH 7.4), containing 0.05% (*w*/*v*) bovine serum albumin (BSA) and then blocked with 5% (*w*/*v*) BSA in the washing buffer for 1 h at room temperature. Next, the replicas were washed and reacted with a mouse monoclonal antibody against the GluN1 subunit of NMDAR (10 μg/ml), diluted in 25 mM TBS containing 1% (*w*/*v*) BSA overnight at 15°C. Following three washes in 0.05% BSA in TBS and blocking in 5% (*w*/*v*) BSA/TBS, replicas were incubated in goat anti‐mouse IgGs coupled to 10 nm gold particles (1:30; British BioCell International, Cardiff, UK) diluted in 25 mM TBS containing 5% (*w*/*v*) BSA overnight at room temperature. When the primary antibody was omitted, no immunoreactivity was observed. After immunogold labelling, the replicas were immediately rinsed three times with 0.05% BSA in TBS, washed twice with distilled water and picked up onto grids coated with pioloform (Agar Scientific, Stansted, Essex, UK).

### 2.5 Quantification and analysis of SDS‐FRL data

The labelled replicas were examined using a transmission electron microscope (JEOL‐1400Flash) equipped with a digital high‐sensitivity scientific Complementary Metal–Oxide–Semiconductor (sCMOS) camera, and images captured at magnifications of 30,000×. The antibody used in this study was visualised by immunoparticles on the exoplasmic face (E‐face), consistent with the extracellular location of the epitopes. Non‐specific background labelling for anti‐GluN1 was estimated by counting immunogold particles on the protoplasmic face (P‐face) surfaces in WT mice. This value was on average 1.2 immunoparticles/μm^2^ and was not subtracted from values for specific labelling, given the low value. Digitised images were then modified for brightness and contrast using Adobe PhotoShop CS5 (Mountain View, CA, USA) to optimise them for quantitative analysis.

#### 2.5.1 Number and density of GluN1 immunoparticles at synaptic and extrasynaptic sites

We determined the number of GluN1 immunoparticles composing excitatory synapses and extrasynaptic membranes of spines and shafts of pyramidal cells and interneuron dendrites located in the stratum oriens (so), stratum radiatum (sr) and stratum lacunosum‐moleculare (slm) of the CA1 field of the hippocampus, in the two genotypes (WT and P301S) at 10 months of age. For that purpose, we used the software Gold Particle Detection and Quantification (GPDQ) developed recently to perform automated and semi‐automated detection of gold particles present in each compartment of neurons [ 27 ]. Most of the spines in the CA1 field arise from pyramidal cells, thus we will refer to them as pyramidal cell spines. Non‐spiny dendrites receiving several synapses, identified as multiple intramembrane particle (IMP) clusters on dendrites, are considered to originate from interneurons.

Quantitative analysis of immunogold labelling for GluN1 was performed on excitatory post‐synaptic specialisations, as well as at extrasynaptic sites, indicated by the presence of IMP clusters on E‐face [ 28 ]. Excitatory post‐synaptic specialisations were considered as such, when IMP clusters contained at least 30 IMPs [ 29 ]; the rest of the neuronal compartment with isolated IMPs was considered as extrasynaptic membrane. In this respect, most of the spines in sr arise from pyramidal cells, thus we refer to them as pyramidal cell spines. Dendritic shafts receiving multiple excitatory and inhibitory synapses are considered to originate from interneurons. For identification of neuronal compartment in the SDS‐FRL samples, oblique dendrites were identified based on their small diameter and the presence of at least one emerging spine from the dendritic shaft. Dendritic spines were considered as such if: (i) they emerged from a dendritic shaft or (ii) they opposed an axon terminal. Dendritic spines are smaller in size compared to dendritic shafts of interneurons. Given these differences in size, excitatory synapses in spines are normally observed with a concave shape, while in interneurons they have a more flattened morphology. Axon terminals were identified based on: (i) the presence of an active zone facing a post‐synaptic density (PSD), recognised by an accumulation of IMPs, on the opposing E‐face of a spine or dendrite; or (ii) the presence of synaptic vesicles on their cross‐fractured portions. One of the advantages of the SDS‐FRL technique is that the whole synaptic specialisation of excitatory synapses and extrasynaptic plasma membrane is immediately visible over the surface of neurons. The outline of post‐synaptic specialisation (IMP clusters) was manually demarcated by connecting the outermost IMP particles, and then we demarcated the rest of the neuronal compartment on the E‐face corresponding to the extrasynaptic plasma membrane. The area of synaptic and extrasynaptic sites was measured using the software GPDQ.

Immunogold particles for GluN1 were regarded as synaptic labelling if they were within the demarcated IMP clusters and those located in the immediate vicinity within 30 nm from the edge of the IMP clusters, given the potential distance between the immunogold particles and antigens. Immunogold particles not meeting those requirements or present in the vicinity of isolated IMPs were regarded as extrasynaptic labelling. The number of immunogold particles was counted in both complete and incomplete (partially fractured) post‐synaptic membrane specialisation. Because densities of immunogold labelling for the GluN1 antibody obtained from complete and incomplete synapses were not significantly different, they were pooled. The density of the immunoparticles for GluN1 in each synaptic site was calculated by dividing the number of the immunoparticles by the area of the demarcated IMP clusters. The density of GluN1 immunoparticles in extrasynaptic sites was calculated by dividing the number of the immunoparticles by the area of the demarcated compartment without the synaptic specialisation. Measurements were performed in three animals, and results were pooled because the average densities for immunogold particles were not significantly different between individual mice in both WT and P301S mice. Immunoparticle densities were presented as mean ± *SEM* between animals.

### 2.6 Controls

To test method specificity in the procedures for electron microscopy, replicas were incubated according to the protocol described above with primary antibodies omitted or replaced with 1% (*v*/*v*) normal goat serum. Labelling densities on clusters of IMPs were <1.2 particles/μm^2^ in these cases.

### 2.7 Data analysis

To avoid observer bias, we performed blinded experiments for immunoblots and immunohistochemistry prior to data analysis. Statistical analyses were performed using GraphPad Prism (San Diego, CA) and data were presented as mean ± *SEM* unless indicated otherwise. Statistical significance was defined as *p* < 0.05. The statistical evaluation of the immunoblots was performed using the Student *t*‐test, with Holm–Sidak method. To compute *SEM* error bars, five blots were measured from each animal. The statistical evaluation of the immunogold densities in the mouse model was performed using the Student *t*‐test, test for homogeneity of variance and Shapiro Wilks normality test, and Mann–Whitney U test for non‐parametric data. Correlations were assessed using Spearman's correlation test.

## 3 RESULTS

### 3.1 Altered expression of NMDARs in the hippocampus of P301S mice

**FIGURE 1:**
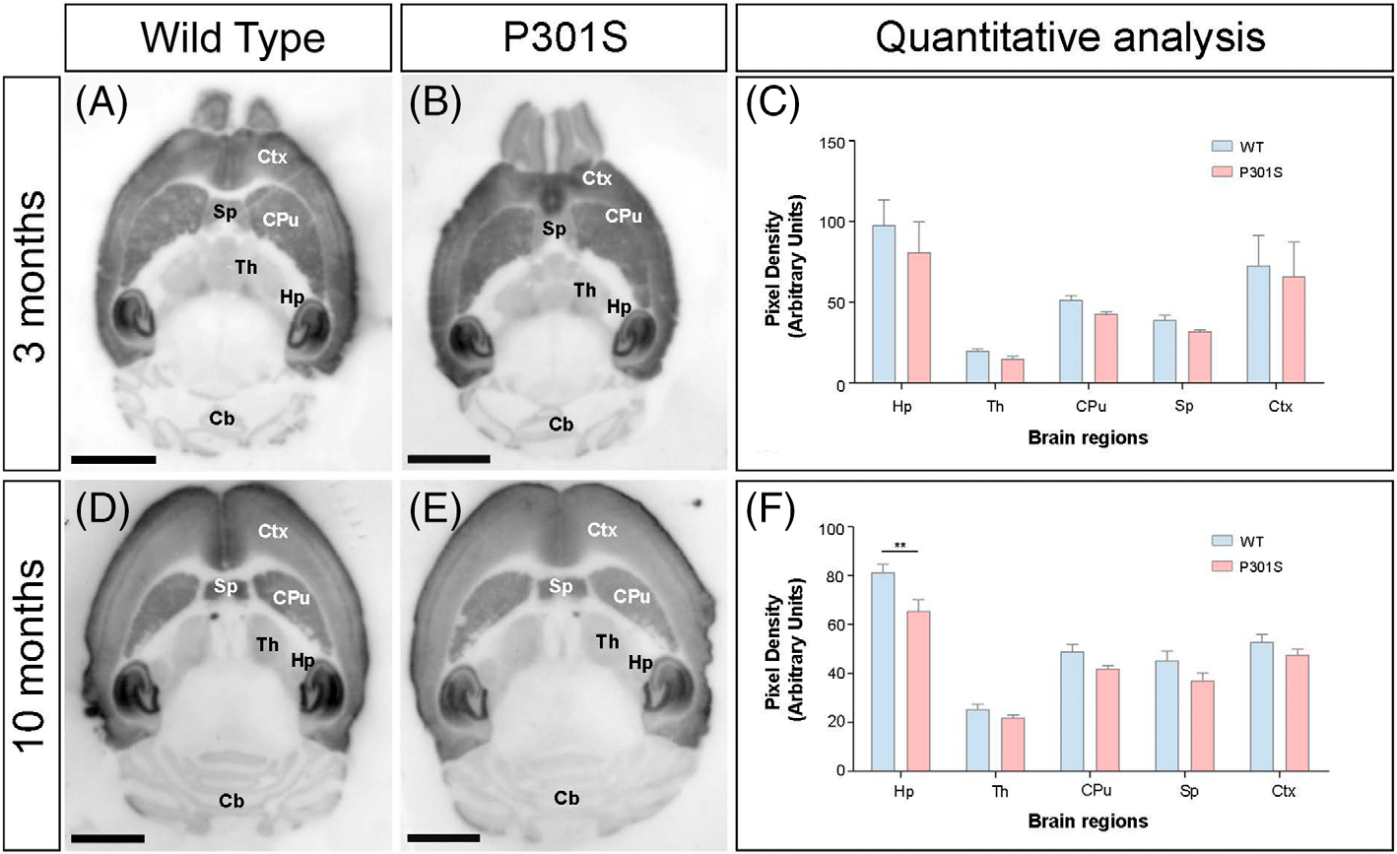
Brain expression of *N*‐methyl‐d‐aspartate receptors (NMDARs) in P301S mice. (A–F) The expression of the GluN1 protein was visualised using histoblots of horizontal brain sections at 3 and 10 months of age in WT and P301S mice using a monoclonal anti‐GluN1 antibody. The expression of NMDARs in different brain regions was determined by densitometric analysis of the scanned histoblots (C and F). The strongest GluN1 expression was detected in the hippocampus (Hp), followed by the cortex (Ctx). Moderate expression levels were detected in the caudate putamen (CPu), the septum (Sp) and the thalamus (Th), and weak in the cerebellum (Cb). Densitometric analysis performed at 3 months revealed no differences in GluN1 expression in P301S mice compared to age‐matched wild‐type controls, but a significant reduction was detected in the hippocampus at 10 months of age (*n* = 3 animals per genotype and per age; Mann–Whitney test, **p < 0.01). Error bars indicate *SEM*. Scale bars: 0.25 cm

Using a monoclonal antibody against the GluN1 subunit, we first established in conventional histoblots [ 24 ] whether the expression of NMDARs was altered in the brain of P301S mice at 3 and 10 months (Figure 1A–F). In WT mice at the two ages, GluN1 immunoreactivity was widely distributed in the brain, with strong labelling in the hippocampus and the neocortex, followed by the caudate putamen and septum (Figure 1A,D). Moderate labelling was found in the thalamus and weak in the cerebellum and midbrain nuclei (Figure 1A,D). Qualitatively, this brain expression pattern was similar in the brain of P301S mice (Figure 1B,E). Quantitative analysis allowed us to compare the protein densities. This revealed that GluN1 immunoreactivities were similar between WT and P301S mice at 3 months (Figure 1C) but showed a significant decrease in labelling in the hippocampus at 10 months of age (Figure 1F). For these data, densitometry in the hippocampus was calculated as a mean value of the three dendritic layers of the CA1 field.

**FIGURE 2:**
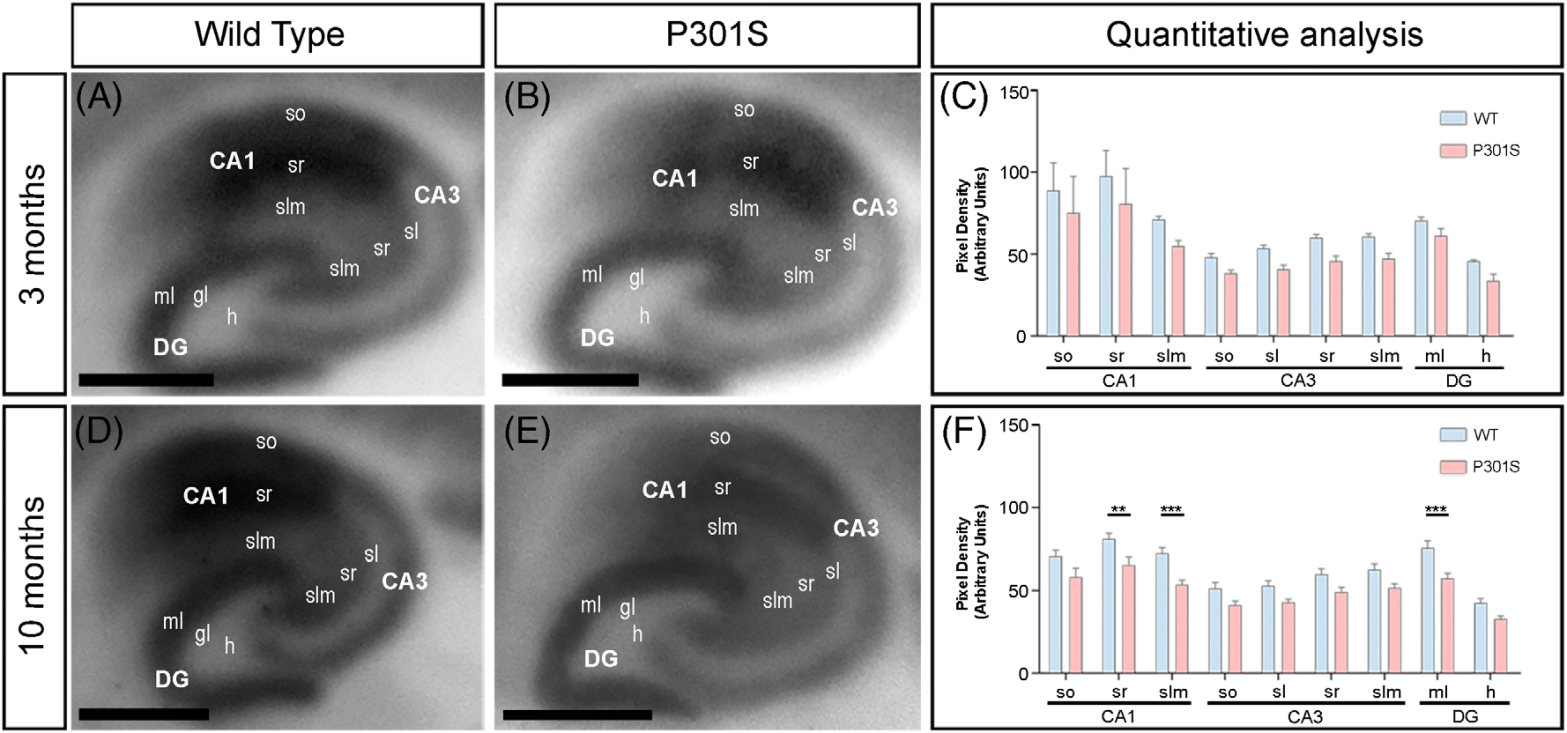
Laminar expression of *N*‐methyl‐d‐aspartate receptors (NMDARs) in the hippocampus of P301S mice. (A–F) The laminar expression of the GluN1 protein in the hippocampus was visualised using histoblots of horizontal sections at 3 and 10 months of age in WT and P301S mice using a monoclonal anti‐GluN1 antibody. The expression of GluN1 in different hippocampal subfields and dendritic layers was determined by densitometric analysis of the scanned histoblots (C and F). The expression of GluN1 was strong in all dendritic layers of the CA1 and CA3 field and dentate gyrus. Densitometric analysis performed at 3 months of age showed no differences in GluN1 expression in P301S mice compared to age‐matched WT controls. However, the expression of GluN1 was significantly reduced in the sr and slm of the CA1 field and the molecular layer of the dentate gyrus of P301S mice compared to age‐matched wild type controls mice at 10 months of age (*n* = 3 animals per genotype and per age; Mann–Whitney U test, **p < 0,01; ***p < 0.001). Error bars indicate *SEM*. CA1, CA1 field of the hippocampus; CA3, CA3 field of the hippocampus; DG, dentate gyrus; gc, granule cell layer; h, hilus; ml, molecular layer; sl, stratum lucidum; slm, stratum lacunosum‐moleculare; so, stratum oriens; sp, stratum pyramidale; sr, stratum radiatum. Scale bars: 0.05 cm

Given the decrease in GluN1 immunolabelling in the hippocampus, we next focused on this brain region to explore laminar expression patterns. Strong GluN1 protein signal was detected in the Ammon's horn and the dentate gyrus (Figure 2A–F). The CA1 field of the Ammon's horn showed the highest GluN1 expression levels in the hippocampus at 3 and 10 months of age (Figure 2A–F). Regarding dendritic layers, GluN1 expression was strong in the so and sr, with the slm showing a moderate expression levels (Figure 2A,B,D,E). The expression levels of GluN1 were moderate in the so, stratum lucidum (sl), sr and slm of the CA3 field (Figure 2A,B,D,E). In the dentate gyrus, GluN1 immunolabelling was moderate to strong in the molecular layer (ml) and weak in the hilus (h) (Figure 2A,B,D,E). Quantitative analyses of immunoreactivities performed at the two ages indicated that the laminar labelling pattern was unchanged in both WT and P301S mice at 3 months of age (Figure 2C). However, the expression of GluN1 was significantly reduced in the sr and slm of the CA1 field and the ml of the dentate gyrus of P301S mice compared to age‐matched WT controls mice at 10 months of age (Figure 2F).

### 3.2 NMDAR content of excitatory synapses is reduced in spines of P301S mice at 10 months

Using the SDS‐FRL technique, we analysed the distribution and densities of GluN1 in different populations of excitatory synapses in the CA1 field: (i) on pyramidal cell spines and (ii) on interneuron dendrites, in so, sr and slm. Replicas obtained from 10 months of age WT and P301S mice were reacted with a monoclonal anti‐GluN1 antibody. Clusters of IMPs on the E‐face represent the post‐synaptic membrane specialisation (PSDs) of glutamatergic synapses [ 30 ]. Only IMP clusters that contained at least 30 IMPs and labelled for GluN1 were regarded as PSDs and included in this analysis.

**FIGURE 3:**
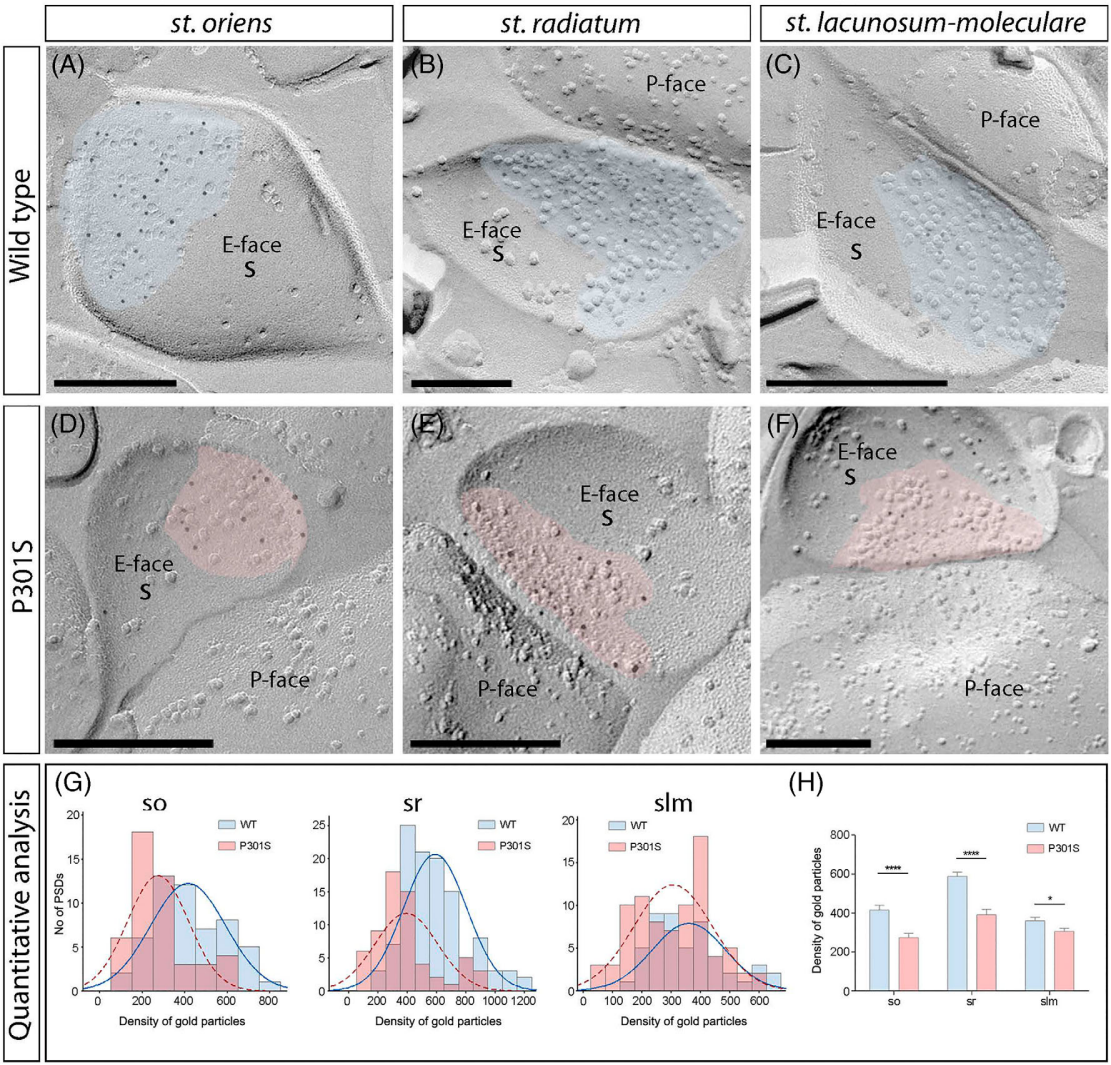
Reduced density of synaptic *N*‐methyl‐d‐aspartate receptors (NMDARs) in spines of P301S mice. (A–F) Electron micrographs of the hippocampus showing immunoparticles for GluN1 at excitatory synaptic sites of spines of pyramidal cells in the three dendritic layers of the CA1 field, as detected using the SDS‐FRL technique in wild‐type and P301S mice at 10 months of age. Post‐synaptic membrane specialisations (IMP clusters, pseudo‐coloured with transparency in blue for wild type and in red for P301S) show strong immunoreactivity for GluN1 (10 nm gold particles) in wild type in strata oriens, radiatum and lacunosum‐moleculare but weaker immunoreactivity in the P301S in the three strata. (G) Differential density of NMDARs in IMP clusters. Box charts showing the distribution of densities of immunoparticles for NMDARs of individual postsynaptic membrane specialisations in the hippocampal CA1 field in wild‐type and P301S mice. (H) Quantitative analysis showing mean densities of NMDARs in excitatory synapses in spines. A significant reduction in the density of NMDAR immunoparticles was detected in spines located in the three strata of the CA1 field of P301S mice compared to age‐matched wild type (*n* = 3 animals per genotype; Mann–Whitney U test, *p < 0.01, ****p < 0.0001). Scale bars: A–F, 200 nm

We initially performed the ultrastructural analysis at excitatory synapses in CA1 pyramidal cell spines, both in WT and P301S mice (Figure 3A–F). In WT mice, immunoparticles for GluN1 were found mainly on excitatory synapses of spines present in the so, sr and slm (Figure 3A–C). In all spines, immunoparticles for GluN1 were randomly distributed over the entire PSDs without forming clusters. In P301S mice, fewer immunoparticles for GluN1 were detected on excitatory synapses of spines in the three CA1 dendritic layers (Figure 3D–F). These possible differences in the content of synaptic NMDARs between the WT and P301S mice were tested (Figure 3G; Table 1). Quantitative analyses revealed that although the density of labelling varied between synapses (Figure 3G,H), there was a significant reduction in GluN1 levels in excitatory synapses on spines in P301S mice (so, 285 ± 23 particles/μm^2^, *n* = 48 synapses; sr, 392 ± 27 particles/μm^2^, *n* = 63 synapses; and slm, 306 ± 15 particles/μm^2^, *n* = 83 synapses) compared to age‐matched WT controls (so, 427 ± 26 particles/μm^2^, *n* = 55 synapses; sr, 590 ± 20 particles/μm^2^, *n* = 112 synapses; and slm, 361 ± 18 particles/μm^2^, *n* = 49 synapses) (Mann–Whitney U test, ****p < 0.0001, **p < 0.01, *p < 0.05) (Figure 3H; Table 1).

**TABLE 1:** Number and density of gold particles for GluN1 at different excitatory synapses in the CA1 region at 10 months of age

	Stratum oriens		Stratum radiatum		Stratum lacunosum‐moleculare	
	Spines	Interneurons	Spines	Interneurons	Spines	Interneurons
WT						
Excitatory synapses (*n*)	55	40	112	60	49	44
Area of synapses (μm)	0.029 ± 0.002	0.049 ± 0.005	0.024 ± 0.001	0.047 ± 0.031	0.027 ± 0.011	0.057 ± 0.036
Median gold particles	10	16	11	17	9	13.5
Range	28–3	34–1	35–2	40–4	19–3	50–6
Density gold particles (μm^2^)						
Mean (±*SEM*)	426.7 ± 25.84	328.6 ± 20.42	590.0 ± 20.46	518.7 ± 27.57	360.7 ± 17.77	249.6 ± 10.72
Median	391.7	327.9	548.8	478.3	325.7	246.4
Range	991.9–90.76	630.0–63.33	1235.27–190.5	1023.26–208.90	656.8–163.0	388.2–120.1
P301S						
Excitatory synapses (*n*)	48	47	63	82	83	38
Area of synapses (μm)	0.024 ± 0.005	0.052 ± 0.015	0.023 ± 0.002	0.049 ± 0.007	0.025 ± 0.002	0.064 ± 0.052
Median gold particles	9	17	7	16	8	14.5
Range	33‐2	70‐4	27‐1	75‐2	34‐2	55‐2
Density gold particles (μm^2^)						
Mean (±*SEM*)	285.4 ± 23.22	280.4 ± 21.98	392.5 ± 27.03	326.9 ± 17.93	306.9 ± 14.70	230.8 ± 14.90
Median	252.0	246.9	346.2	297.4	324.1	245.5
Range	798.7–64.08	837.7–62.46	942.4–57.82	837.2–36.41	606.6–39.76	394.7–56.43

*Note*: Density values are provided in immunogold/square micrometer.

**FIGURE 4:**
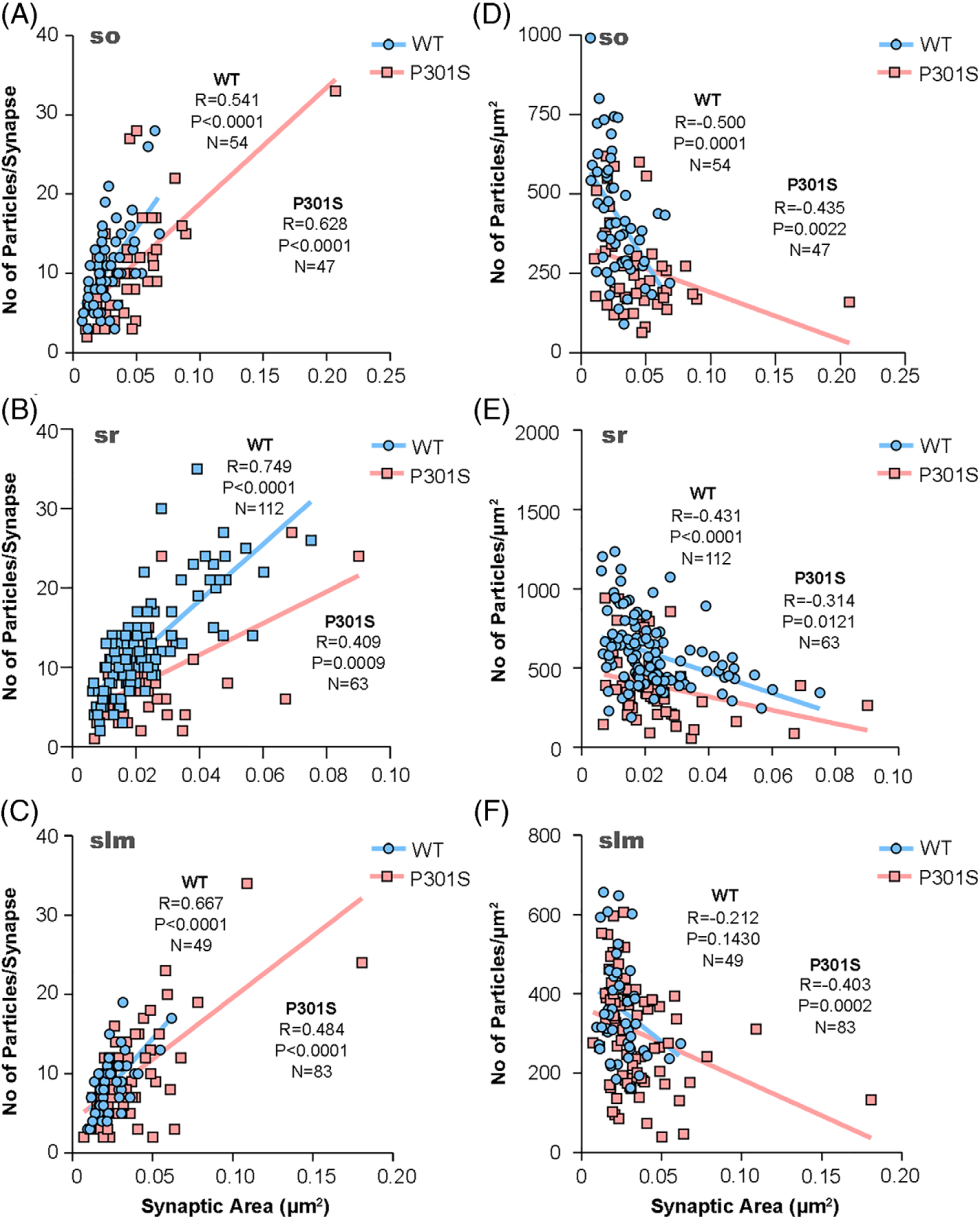
*N*‐methyl‐d‐aspartate receptor (NMDAR) immunoparticle density at excitatory synapses on spines. Correlation of the number of GluN1 immunoparticles and IMP‐cluster area on pyramidal cell spines. (A–C) Scatterplots of the number of immunoparticles for GluN1 versus the size of excitatory synapses in the three dendritic layers in both wild type and P301S mice. In all cases, there is a positive linear correlation between immunoparticle number and synaptic size (Spearman's rank‐order correlation). (D–F) Comparison of the relationships between density for GluN1 and size of excitatory synapses in the three dendritic layers in both wild type and P301S mice. The immunoparticle density for GluN1 is negatively correlated with synapse size (Spearman's rank‐order correlation).

The size of the excitatory synapses established on spines in the three layers of the CA1 field revealed no significant differences between WT and P301S mice (Mann–Whitney *U* test, *p* > 0.1) (Table 1). The number of GluN1 immunoparticles in those synapses was highly variable but consistently lower in P301S mice (Table 1), although linear correlation between the number of immunoparticles and area of synaptic sites was found for both mouse genotypes (i.e., WT and P301S) (Figure 4A–F; Table 1), indicating a uniform receptor density across all excitatory synapses in normal and pathological conditions. Furthermore, the density of immunoparticles for GluN1 per synapse declined with increasing synaptic area (Figure 4A–F), as indicated by the weak negative correlation of immunoparticle density and synaptic area.

### 3.3 Differential reduction of NMDARs in excitatory synapses in interneurons of P301S mice

**FIGURE 5:**
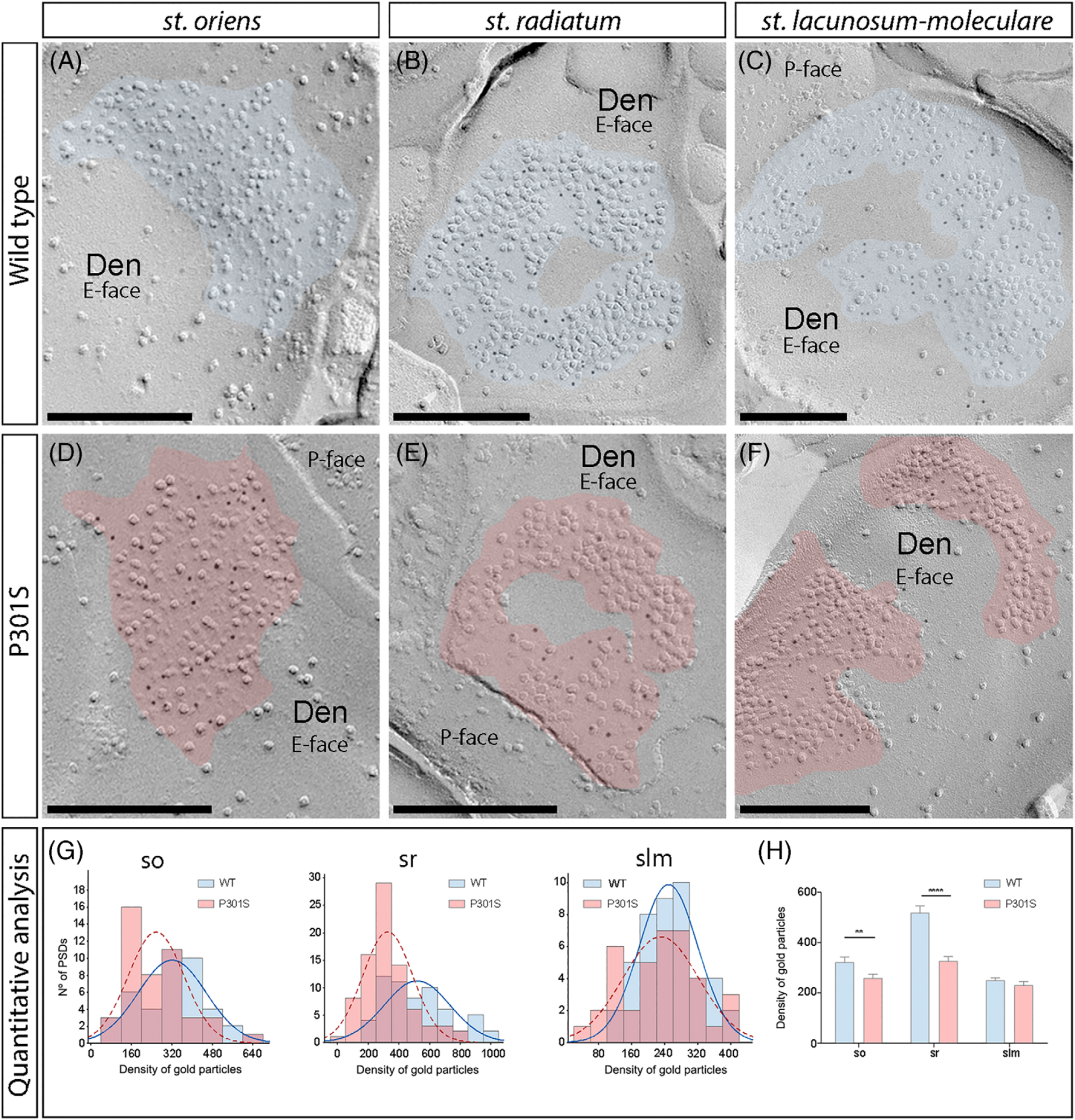
Density of synaptic *N*‐methyl‐d‐aspartate receptors (NMDARs) in interneurons of P301S mice. (A–F) Electron micrographs of the hippocampus showing immunoparticles for GluN1 at excitatory synaptic sites of interneuron dendrites in the CA1 field, as detected using the SDS‐FRL technique in P301S mice at 10 months of age. Postsynaptic membrane specialisations (IMP clusters, pseudo‐coloured with transparency in blue for wild type and in red for P301S) on interneuron dendrites show strong immunoreactivity for GluN1 (10 nm gold particles) in the wild type. In P301S mice, lower number of GluN1 immunoparticles in the P301S in strata oriens, radiatum and lacunosum‐moleculare. (G) Box charts showing the distribution of densities of immunoparticles that label NMDARs of individual post‐synaptic membrane specialisations in the hippocampal CA1 field in wild type and P301S mice. (H) Quantitative analysis showing mean densities of NMDARs in excitatory synapses in interneuron dendrites. A significant reduction in the density of NMDAR immunoparticles was detected in interneuron dendrites distributed in the strata oriens and radiatum, but no changes were found in the stratum lacunosum‐moleculare of the CA1 field of P301S mice compared to age‐matched wild type (Mann–Whitney U test, **p < 0.01, ****p < 0.0001). Scale bars: A–F, 200 nm

We next analysed qualitatively the distribution and densities of GluN1 in excitatory synapses on interneuron dendrites in the CA1 field using the SDS‐FRL technique in both WT and P301S mice of 10 months old (Figure 5A–F). The majority of immunoparticles for GluN1 were distributed over the entire post‐synaptic membrane specialisations with no apparent clustering (Figure 5A–C). In P301S mice, immunoparticles for GluN1 were also randomly distributed over the entire post‐synaptic membrane specialisations in the so, sr and slm but detected at a lower frequency than in WT mice (Figure 5D–F).

We quantified the changes in the density of GluN1 in excitatory synapses on interneuron dendrites between the two experimental groups (Figure 5G,H; Table 1). In WT mice, the density of labelling varied from synapse to synapse in the three dendritic layers, with mean values of 329 ± 20 particles/μm^2^ (so, *n* = 40 synapses), 519 ± 28 particles/μm^2^ (sr, *n* = 60 synapses), and 250 ± 11 particles/μm^2^ (slm, *n* = 44 synapses) (Figure 5H; Table 1). In P301S mice, the density of GluN1 immunoparticles was 280 ± 22 particles/μm^2^ (so, *n* = 47 synapses), 327 ± 18 particles/μm^2^ (sr, *n* = 82 synapses) and 231 ± 15 particles/μm^2^ (slm, *n* = 38 synapses) (Figure 5H; Table 1). Comparisons between the density of labelling in all examined interneuron dendrites showed that there is a significant reduction of GluN1 in excitatory synapses in the so and sr (Mann–Whitney U test, **p < 0.01, ****p < 0.0001) but not in the slm (Mann–Whitney U test, *p* > 0.05) (Figure 5H; Table 1), indicating the existence of a differential reduction in the synaptic localisation of NMDARs in interneurons in P301S mice.

**FIGURE 6:**
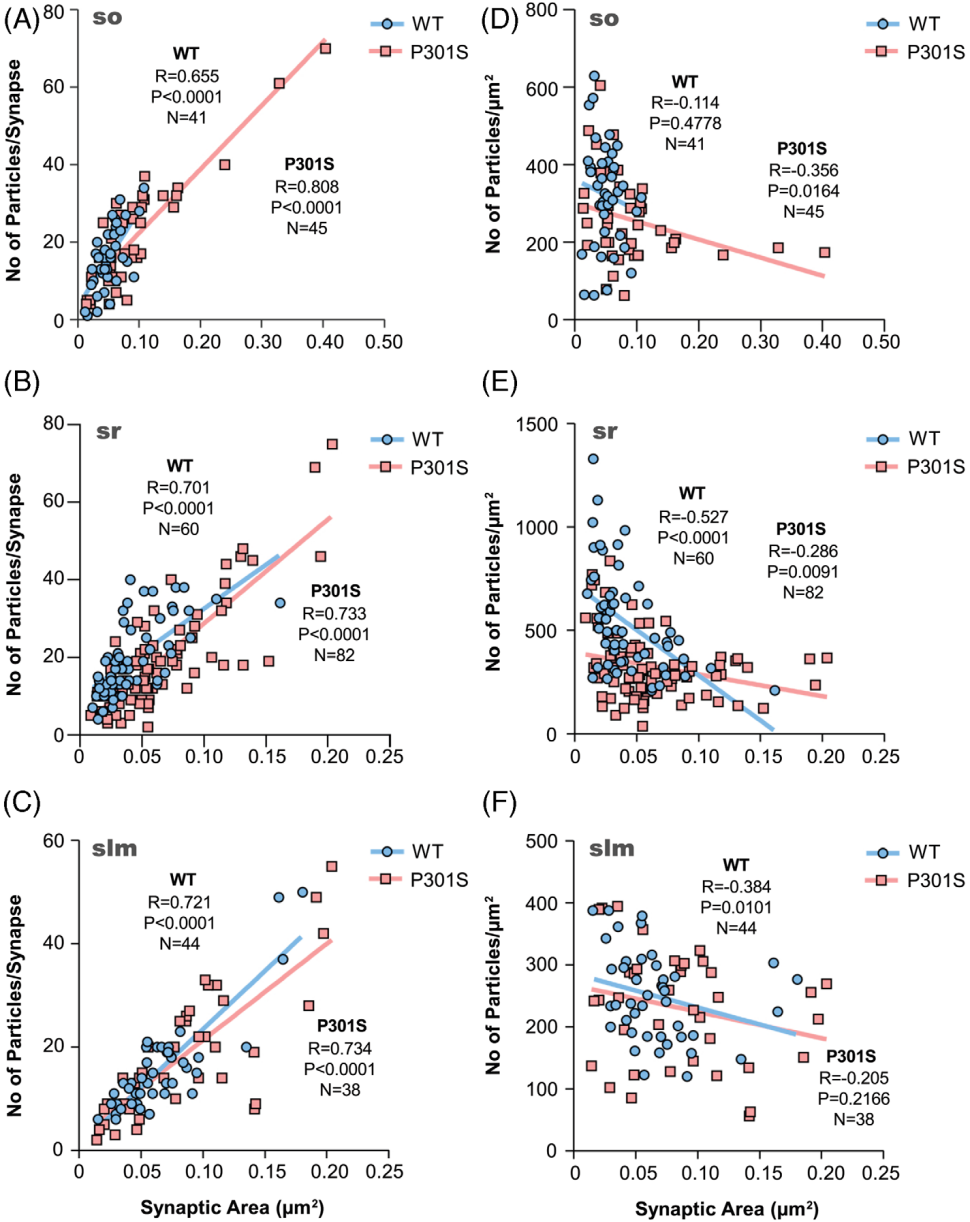
*N*‐methyl‐d‐aspartate receptor (NMDAR) immunoparticle density at excitatory synapses on interneurons. Correlation of the number of GluN1 immunoparticles and IMP‐cluster area on interneuron dendrites. (A–C) Scatterplots of the number of immunoparticles for GluN1 versus size of excitatory synapses on interneuron dendrites in both wild type and P301S mice. In the three dendritic layers, there is a positive linear correlation between immunoparticle number and synaptic size (Spearman's rank‐order correlation). (D–F) Comparison of the relationships between density for GluN1 and size of excitatory synapses in the three dendritic layers in both wild type and P301S mice. In synapses of interneuron dendrites, there is no correlation between the parameters, except in wild‐type mice in the *stratum radiatum* (Spearman's rank‐order correlation).

The size of the excitatory synapses established on interneuron dendrites in the so, sr and slm revealed no significant differences between WT and P301S mice (Mann–Whitney *U* test, *p* > 0.05). The number of GluN1 immunoparticles in those synapses was highly variable in both WT and P301S mice (Table 1), but linear correlation between the number of immunoparticles and area of synaptic sites was found both in WT and P301S mice (Figure 6A–F; Table 1). This indicated a uniform receptor density across excitatory synapses established on interneuron dendrites in normal and pathological conditions. Furthermore, a weak negative correlation between the size of synapses and the density of immunogold particles was found (Figure 6A–F), indicating that the density of immunoparticles for GluN1 per synapse declined with increasing synaptic area in interneurons (Spearman's correlation test).

### 3.4 Extrasynaptic NMDAR content in the hippocampus of P301S mice

**FIGURE 7:**
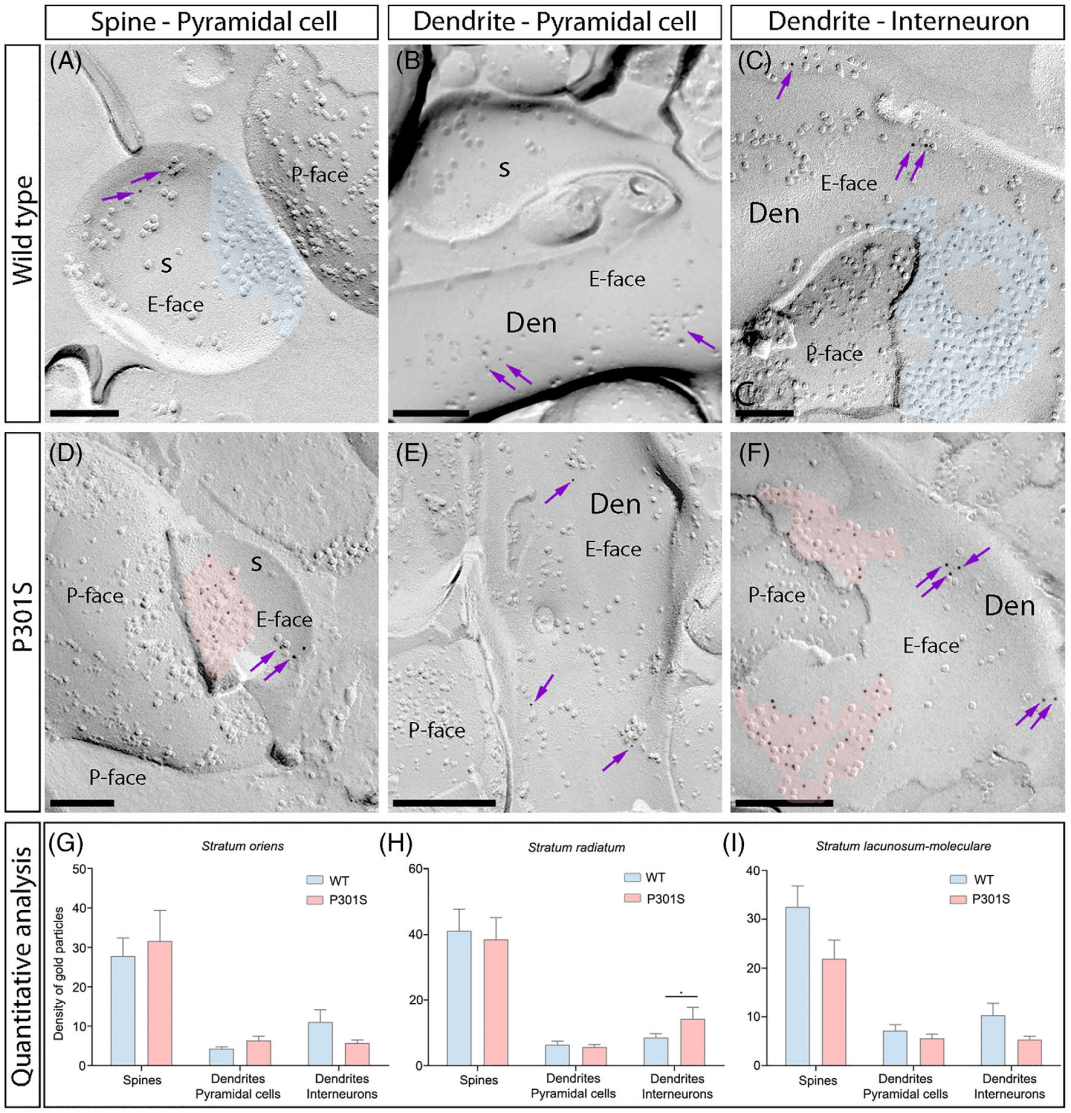
Density of extrasynaptic NMDARs in the hippocampus of P301S mice. (A–F) Electron micrographs of the hippocampus showing immunoparticles for GluN1 at extrasynaptic sites of spines and dendrites of pyramidal cells and interneuron dendrites in the CA1 field, as detected using the SDS‐FRL technique in P301S mice at 10 months of age. For illustration purposes, these electron micrographs are only from the sr. Extrasynaptic membrane compartments showed only very weak immunolabelling for GluN1, with isolated immunoparticles (10 nm gold particles) associated with IMPs in both wild type and P301S mice. Post‐synaptic membrane specialisations representing synaptic sites are pseudo‐coloured with transparency in blue for wild type and in red for P301S. (G–I) Quantitative analysis showing mean densities of NMDARs in extrasynaptic compartments in the three dendritic layers of the CA1 field. A significant increase in the density of GluN1 immunoparticles at extrasynaptic sites was detected in interneuron dendrites distributed in the sr of P301S mice compared with age‐matched wild type (Mann–Whitney U test, *p < 0.05). No differences were detected in all other compartments throughout the CA1 field (Mann–Whitney U test, *p* > 0. 05). Scale bars: A–F, 200 nm

0.005w?>In addition to their synaptic localisation, NMDARs are also found at lower density at extrasynaptic sites [ 21 ]. To investigate whether extrasynaptic NMDARs undergo changes in pathological conditions, we analysed the distribution and densities of GluN1 in the extrasynaptic membranes of pyramidal cells and interneuron dendrites in the three layers of the CA1 field. Only isolated IMPs or IMP clusters that contained >30 IMPs and labelled for GluN1 were regarded as extrasynaptic and included in the analysis. Immunoparticles for GluN1 were observed at a low frequency and randomly distributed over the extrasynaptic plasma membrane as isolated gold particles in both WT and P301S mice (Figure 7A–F).

When the density of extrasynaptic GluN1 labelling was analysed in spines and shafts of pyramidal cells and interneuron dendrites throughout the so, sr and slm, we found no differences between WT and P301S mice in most compartments (Mann–Whitney U test, *p* > 0.05) (Figure 7G–I, Table 2). However, we detected a significant increase of extrasynaptic GluN1 in the dendrites of interneurons in the sr of P301S mice (14.2 ± 3.6 particles/μm^2^) compared to age‐matched WT (8.6 ± 1.2 particles/μm^2^) (Mann–Whitney U test, *p < 0.05) (Figure 7H, Table 2). In summary, contrary to the reduction for synaptic NMDARs, these results indicate that extrasynaptic NMDARs are mostly unaltered in WT and P301S mice at 10 months of age, and when altered they are significantly increased.

**TABLE 2:** Number and density of gold particles for GluN1 at extrasynaptic compartments in the CA1 region at 10 months of age

	Stratum oriens			Stratum radiatum			Stratum lacunosum‐moleculare		
	PC spines	PC dendrites	Interneurons	PC spines	PC dendrites	Interneurons	PC spines	PC dendrites	Interneurons
WT									
Extrasynaptic compartments (*n*)	21	21	26	37	36	56	29	32	26
Density gold particles (μm^2^)									
Mean (±*SEM*)	27.8 ± 4.61	4.3 ± 0.49	11.10 ± 3.09	41.10 ± 6.71	6.31 ± 1.17	8.64 ± 1.19	32.51 ± 4.32	7.21 ± 1.20	10.34 ± 2.48
Median	23.44	4.09	4.92	29.03	4.65	6.23	28.45	4.77	3.78
Range	6.36–87.63	1.32–9.19	1.32–68.66	6.10–208.8	0.84–42.09	1.23–52.68	6.46–105.2	0.97–28.63	0.85–44.39
P301S									
Extrasynaptic compartments (*n*)	20	26	38	25	24	60	21	27	38
Density gold particles (μm^2^)									
Mean (±*SEM*)	31.61 ± 7.80	6.39 ± 1.08	5.74 ± 0.77	38.53 ± 6.72	5.71 ± 0.80	14.25 ± 3.58	21.87 ± 3.78	5.60 ± 0.88	5.36 ± 0.69
Median	19.58	5.40	4.05	24.25	4.27	7.91	17.69	3.29	4.33
Range	6.82–161.9	1.3–27.56	0.97–25.34	9.15–143.2	1.69–17.74	1.66–210.3	4.39–86.72	1.28–16.75	0.83–19.66

*Note*: Density values are provided in immunogold/square micrometer.

Abbreviation: PC, pyramidal cell.

## 4 DISCUSSION

Activation of NMDARs in the hippocampus provides a major fast excitatory pathway that plays an essential role in synaptic mechanisms of learning and memory [ 31 ]. Several lines of evidence suggest a role for an altered NMDAR function in the pathogenesis of chronic neurodegenerative disorders including AD (reviewed by Wang and Reddy [ 2 ]), and consequently these receptors are currently viewed as valuable therapeutic targets in this disorder [ 32, 33 ]. However, despite their obvious importance in pathological conditions, no information is yet available whether the expression and subcellular distribution of NMDARs undergo changes in transgenic models of AD. In our work, we sought to investigate whether hyperphosphorylation of tau protein, a pathological hallmark of AD, is associated with alterations in the expression and content of NMDARs. Given that all NMDARs contain the obligatory GluN1 subunit, receptor expression and localisation were performed with an antibody recognising all splice variants of GluN1. To compare the NMDAR content in normal and pathological conditions, we employed the high‐resolution SDS‐FRL technique combined with quantitative approaches and analysed the excitatory synapses and extrasynaptic sites on pyramidal cell spines and interneuron dendrites in the CA1 field of the hippocampus in WT and P301S mice. Our data suggest that tau toxicity impacts differentially on both synaptic and extrasynaptic receptors, driving robust decline of NMDARs in excitatory synapses on different classes of neurons in P301S mice. These alterations may be involved in the mechanisms causing abnormal network activity of the hippocampal circuit as described in this animal model of AD.

### 4.1 Expression profile of NMDARs in P301S mice

The GluN1 subunit of NMDARs is expressed strongest in the CA1 field of the adult hippocampus [ 8, 34, 35 ]. Accordingly, we have shown by histoblot that GluN1 was widely expressed in the hippocampus at 3 and 10 months old and the labelling was particularly strong in dendritic layers of CA1, where excitatory synapses are established in spines and interneuron dendrites. However, little is known about the expression of GluN1 in animal models of AD. The present work revealed that the overall brain expression pattern of GluN1 protein is not the same in all regions, with the more prominent changes occurring in the hippocampus. We observed a significant decrease in the protein expression of GluN1 in 10 monthsold P301S mice. This is consistent with the findings described in rTg4510 mice [ 36 ], in tau‐transfected neurons [ 37 ] and in neuronal cultures overexpressing P301L tau [ 15 ]. Extrapolation of these data obtained in animal models to humans is a difficult task because of the molecular and structural differences between both brains, and because animal models do not reproduce all features of AD as found in humans. However, previous autoradiographic studies showed that binding of glutamate to the NMDAR was significantly decreased, particularly in the CA1 field [ 38, 39, 40, 41, 42 ], and immunoblot, reverse transcription‐polymerase chain reaction and immunohistochemical approaches have also described a reduction in the GluN1 protein expression in AD brains [ 43, 44, 45, 46 ], consistent with the data as described in our study.

### 4.2 Reduction of synaptic NMDAR content of pyramidal cells in P301S mice

Pyramidal cells of the CA1 field express mRNA for the GluN1 subunit of the NMDAR in the hippocampus [ 8, 34, 35, 47 ]. Previous studies using immunogold techniques showed that virtually all synapses on the spines of CA1 pyramidal cells contained NMDARs [ 19, 48, 49 ], and reported that the degree of immunolabelling correlated weakly with the size of synapses [ 18, 19 ], which contrasts with the α‐amino‐3‐hydroxy‐5‐methyl‐4‐isoxazolepropionic acid receptor (AMPAR) content [ 49, 50 ]. Our direct measurement using a different immunoelectron microscopic approach confirmed that the NMDAR content of spine synapses correlates with synaptic area in normal and pathological conditions. This correlation is weaker than as described for AMPARs using similar approaches [ 51 ]. Consistent with these findings, we also confirmed that as a result of the weak correlation of synapse size and GluN1 density, the larger the synapse the lower the density of NMDARs on spines [ 18, 19 ]. Such relationships between synapse size and receptor numbers apply to the three dendritic layers both in WT and P301S mice. Given that spines throughout the CA1 field receive anatomically segregated glutamatergic inputs [ 52 ], the significant decline in NMDAR densities in P301S mice suggests that tauopathy has similar influence or toxicity on synaptic receptors present in spines facing presynaptic axons, which have originated from different sources.

Although behavioural and electrophysiological deficits have been reported in P301S mice, accompanied by a synapse loss detected using immunohistochemical staining of synaptic markers [ 22, 53, 54, 55 ], studies using microinjection of Lucifer yellow with confocal laser scanning microscopy and neuronal three‐dimensional reconstructions yielded no robust differences in spine density in the CA1 field of the hippocampus [ 56 ]. Despite any possible unaltered spine numbers, P301S mice exhibit deficits in synaptic function, including impaired long‐term potentiation in the CA1 field [ 22 ]. One possible explanation is that pathologic tau accumulation drives functional weakening of receptor channel activity at excitatory synapses before the eventual loss of spines. Consistent with this idea, immunoEM of hyperphosphorylated tau in the hippocampal CA1 field revealed accumulation of tau at synapses, both at pre‐ and post‐synaptic sites, parallel to molecular changes in PSDs of excitatory synapses in the hippocampus [ 57 ]. These studies suggested a toxic effect for tau on excitatory synapses, and supporting this scenario, our data demonstrated a significant decrease in NMDARs in all spines. The exact molecular mechanism for such reduction remains unclear but likely affects the trafficking of receptors and their anchoring proteins. Thus, hyperphosphorylated tau reduces the trafficking of glutamate receptor subunits GluA1 and GluA2/3 to PSD‐95 [ 15 ]. In addition, a mimic of phosphorylated tau impairs post‐synaptic function through the disruption of synaptic trafficking or anchoring of AMPARs and NMDARs in cultured mouse neurons [ 15 ]. Overall, our data strongly support the accumulating evidence suggesting that tau induces synaptic dysfunction [ 37, 58, 59 ] through the decline of NMDARs.

### 4.3 Differential reduction of synaptic NMDARs in interneurons of P301S mice

The role of NMDARs in the signalling, plasticity and excitability of interneurons is less understood. Unlike pyramidal cells, interneurons in the hippocampus possess a rich cellular diversity and include several populations that can be classified based on their synaptic target selectivity, electrophysiological properties and neurochemical features [ 60, 61, 62 ], and functional NMDARs have been shown in most interneuron subtypes [ 60 ]. NMDAR‐mediated responses in interneurons are dependent on the cell type [ 63 ], and all major interneurons subtypes express mRNA for GluN1 and GluN2A/B/D subunits [ 8, 62 ], although some interneurons seem to lack an NMDAR component of the excitatory postsynaptic potential (EPSC) [ 64 ]. Our results demonstrate that excitatory synapses showed a large heterogeneity of synaptic GluN1 content amongst interneuron dendrites, but some synapses contained more GluN1 immunoparticles than the most labelled excitatory synapses on spines. In addition, NMDARs show a lower degree of size‐dependent variability than spines. These findings are consistent with earlier studies using post‐embedding quantitative immunogold [ 18, 19 ] and are also in line with the high variability in the NMDA component as reported in electrophysiological studies [ 64, 65, 66 ].

Several lines of evidence suggest that the dysfunction of interneurons is a major factor causing network alterations associated with cognitive deficits in AD [ 67, 68, 69, 70, 71, 72, 73 ]. Consistent with this GABAergic loss of function, convincing evidence demonstrates the distinct vulnerability and selective degeneration of different interneuron populations in both animal models and human tissue from AD patients [ 74, 75, 76, 77 ]. An interesting finding of our study is that synaptic GluN1 in interneuron dendrites shows different vulnerability to tauopathy injury. We demonstrated that NMDARs are significantly decreased in excitatory synapses established on interneurons dendrites in the so and sr of the hippocampus, but no notable alterations were observed in the slm. These differing observations may represent cell type‐ and layer‐specific differences in response to tau accumulation and may be dependent on the NMDAR subunit composition. In normal conditions, recent reports have established that synaptic function depends on the GluN2 subunit and the interneuron type [ 78 ]. Further studies evaluating the contribution and alteration GluN2A‐D subunits in interneurons in tauopathy are required. Regardless of the role played by GluN2, any loss of GluN1 as described in findings from our work will result in the removal of the inhibitory force, thus potentially increasing the excitotoxic effects of NMDAR activation and causing hyperactivity through disinhibition. In agreement with our finding, previous studies have also demonstrated that disruption to inhibitory cells underlies hyperactivity in mouse models of AD [ 68, 69, 79, 80 ], as well as in AD patients with mild cognitive impairment [ 81, 82, 83, 84 ].

### 4.4 Extrasynaptic NMDAR content in pyramidal cells and interneurons in P301S mice

NMDARs are not only found at synaptic sites but also at extrasynaptic locations [ 21 ]. It is widely accepted that the subcellular localisation of NMDARs determines their physiological roles. Consistent with this idea, synaptic NMDARs tend to promote synaptic plasticity and neuronal survival, while extrasynaptic NMDARs promote neuronal death, synaptic plasticity failure and memory loss [ 85, 86, 87 ]. In addition to their high density at excitatory synapses, an interesting finding of our study is that immunoparticles for GluN1 were also detected at extrasynaptic membrane compartments, although at low densities, in both pyramidal cells and interneurons, in agreement with previous studies using post‐embedding immunogold techniques [ 20, 88 ]. This extrasynaptic labelling is significantly higher than background labelling. More importantly, our quantitative analysis in P301S mice showed that, contrary to the reduction of synaptic NMDARs, labelling density for GluN1 at extrasynaptic sites was unaltered in pyramidal cells and interneurons, but an increased density in interneuron dendrites of the sr was observed when comparing WT with P301S mice. Consistent with this effect of the occurrence of hyperphosphorylated tau, recent studies in tau knockout mice have demonstrated that the absence of tau leads to a decrease in functional extrasynaptic NMDA receptors in the hippocampus [ 89 ].

Overall, our data identified a shift in the balance from synaptic towards extrasynaptic NMDARs. While many extrasynaptic NMDARs may be in transit to/or from synapses acting as a reserve pool for synaptic receptors, the presence of many immunoparticles far away from excitatory synapses suggest that many receptors are in extrasynaptic sites for specific functions [ 90 ]. The distribution of synaptic versus extrasynaptic NMDARs may be an important parameter that determines the susceptibility of neurons to toxic insults. The unbalance between synaptic versus extrasynaptic NMDARs described in our work may be involved in promoting neuronal death effect associated with tau accumulation under pathological conditions. Therefore, therapeutic targeting of extrasynaptic NMDARs could be a promising strategy.

In summary, our study is the first to identify and visualise a synaptic decline of NMDARs on both pyramidal cells and interneurons in tau‐associated pathology. Tau toxicity predominantly impacts excitatory synapses on pyramidal cells, as well as many excitatory synapses on interneurons. This could cause changes in the balance between excitatory and inhibitory neurotransmission leading to abnormal network activity of the hippocampal circuit. The combination of such synaptic failures with the disruption in the synaptic/extrasynaptic balance is likely to be key contributors to the cognitive dysfunctions associated with the P301s mouse model. Therefore, our findings provide new therapeutic opportunities, focused on enhancing neuronal inhibition and targeting of extrasynaptic NMDARs, which may lead to modification of disease progression.

## AUTHOR CONTRIBUTIONS

All authors had full access to all data in the study and take responsibility for the integrity of the data and the accuracy of the data analysis. Rafael Luján and Yugo Fukazawa designed the project. Rocío Alfaro‐Ruiz performed SDS‐FRL immunoelectron microscopy. Rocío Alfaro‐Ruiz, Carolina Aguado and Ana Esther Moreno‐Martínez performed histoblot analysis. Jesús Merchán‐Rubira managed the colony of P301S transgenic mice. Jesús Avila and Félix Hernández provided transgenic mice and feedback on the analysis and manuscript. Rocío Alfaro‐Ruiz, Alejandro Martín‐Belmonte, Carolina Aguado and Rafael Luján analysed data. Rafael Luján wrote the paper. All authors read and approved the final manuscript.

## CONFLICT OF INTEREST

The authors of this manuscript declare that they have no competing interests.

## ETHICAL APPROVAL AND CONSENT TO PARTICIPATE

All animal experimental procedures were performed in accordance with Spanish (RD 1201/2015) and European Union regulations (86/609/EC), and the protocols were approved by the local Animal Care and Use Committee.

## CONSENT FOR PUBLICATION

All co‐authors of the present manuscript can certify that it has not been submitted to more than one journal for simultaneous consideration and that the manuscript has not been published previously (partly or in full). The authors also can certify that our main study is not split up into several parts to increase the quantity of submissions, that none of the data presented here have been fabricated or manipulated and that we present our own data/text/theories/ideas. All authors and authorities have explicitly provided their consent to submit the present manuscript and in general we all agree with the ethical responsibilities of authors of the journal. Finally, all authors give consent for publication in *Brain Pathology*.

## Data Availability

All data used and/or analysed during the current study are available from the corresponding author on reasonable request.
